# Profiling the lncRNA-miRNA-mRNA interaction network in the submandibular gland of diabetic mice

**DOI:** 10.1186/s12902-022-01019-1

**Published:** 2022-04-21

**Authors:** Xi-Jin Shi, Hui-Min Liu, Li Li, Yan Zhang, Xin Cong, Li-Mei Liu, Li-ling Wu, Ruo-Lan Xiang

**Affiliations:** Department of Physiology and Pathophysiology, Peking University School of Basic Medical Sciences, No.38 Xueyuan Road, Haidian District, Beijing, 100191 China

**Keywords:** Diabetes mellitus, Hyposalivation, lncRNA, ceRNA, Microarray

## Abstract

**Background:**

Hyposalivation is one of the common symptoms of diabetes. Although long non-coding RNAs (lncRNAs) have recently been reported to play important roles in the pathogenesis of diabetes, the role of lncRNAs in diabetes-induced hyposalivation remains unknown.

**Methods:**

The present study aimed to explore the function of lncRNA-microRNA-mRNA regulatory network in the submandibular gland (SMGs) under the context of diabetes. LncRNA expression profile of the SMGs was analyzed using microarray technology. Differentially expressed lncRNAs were confirmed using real-time quantitative PCR. Bioinformatics analyses were performed, and Coding-non-coding gene co-expression (CNC) and competing endogenous RNA (ceRNA) networks were constructed to explore the potential mechanisms of diabetes-induced hyposalivation.

**Results:**

A total of 1273 differentially expressed lncRNAs (536 up-regulated and 737 downregulated) were identified in the SMGs tissues of db/db mice. CNC and ceRNA network analyses were performed based on five differentially expressed lncRNAs validated by real-time quantitative PCR. Gene Ontology analysis of target genes of CNC network revealed that “calcium ion binding” was a highly enriched molecular function. Kyoto Encyclopedia of Genes and Genomes pathway analysis of target genes of ceRNA network revealed that the “mammalian target of rapamycin signaling pathway” was significantly enriched.

**Conclusions:**

On the whole, the findings of the present study may provide insight into the possible mechanism of diabetes-induced hyposalivation.

**Supplementary Information:**

The online version contains supplementary material available at 10.1186/s12902-022-01019-1.

## Introduction

Diabetes is a group of metabolic diseases characterized by abnormal insulin secretion and/or insulin resistance. In addition to causing long-term damage to different organs, diabetes can also change the function of salivary glands and may cause changes in the composition and volume of salivary secretion, thus affecting the oral homeostasis [1]. Some studies have found that the salivary flow of diabetic patients is significantly decreased compared with control group [2, 3]. Clinical studies showed that 92.5% of the elderly patients with type 2 diabetes suffered decreased salivary flow rate [4]. Saliva played a key role in digestion, taste, cleaning, hydration of oral mucosa and tooth protection and was essential for maintaining the dynamic balance of the oral environment [5]. Damage to the salivary glands was typically manifested as a reduction in salivary flow, which can be converted into bad subjective feelings such dry mouth, taste disorders, difficulty with swallowing and chewing, and increased risk of caries. These feelings ultimately have a negative impact on quality of life. Many studies have demonstrated that the incidence of oral fungal and bacterial infection, lichen planus and caries was high in patients with diabetes [2, 3, 6]. In our previous study, we observed atrophy of the acini, and decreased stimulated salivary flow of SMG in db/db mice [7]. However, the mechanism by which diabetes induces SMG damage is not clear.

LncRNA is a class of RNA molecules that are greater than 200 nucleotides in length. In recent decades, lncRNAs have been identified as important epigenetic factors that regulate various human diseases, such as cancer, neurodegenerative diseases and cardiovascular diseases [8–12]. Recent studies have shown that the incidence and development of type 2 diabetes was associated with lncRNAs. Downregulation of the lncRNA ANRIL improved the cardiac function index and inflammatory factor expression in diabetic rats, enhanced the pathological state of myocardial tissue and myocardial remodeling, and reduced the area of myocardial collagen deposition [13]. The lncRNA NONRATT021972 was upregulated in diabetic rat nervous system cells, suggesting that it may participate in the pathophysiological processes associated with sympathetic neurons in diabetes [14]. Downregulation of the lncRNA SOX2OT regulated the NRF2/HO-1 signaling pathway and protected against high glucose-induced damage in retinal ganglion cells in vivo and in vitro [15]. Based on these findings, we hypothesized that lncRNAs represented a potential cause of diabetes-induced reductions in salivary secretion. CeRNA can regulate each other at post-transcription level by competing for common miRNAs [16]. LncRNA MEG3 acted as a ceRNA of miR-214 to promote hepatic insulin resistance by facilitating the expression of ATF4 [17]. Protein-coding mRNA DKK1 and PTEN served as ceRNA, showing crosstalk and affecting the expression of each other via competition for miRNAs binding [18]. In studies exploring lncRNAs, the identification of a target gene and its correlation with diabetes-related xerostomia can help us to understand the pathology of diabetes-related xerostomia and provide further information for the treatment of diabetes-related xerostomia.

To better understand the function of lncRNA in the impairment of salivary function in diabetes, this study characterized the expression profiles of lncRNA in the SMG tissues of db/db and db/m mice using microarray. The establishment of CNC and ceRNA networks based on lncRNA may help to explore the potential functions of lncRNAs in diabetes-induced SMG dysfunction.

## Material and methods

### Animal models

Sixteen-week-old male db/db mice (a model of spontaneous type 2 diabetes mellitus) were purchased from Changzhou Cavens Laboratory Animal (Changzhou, China). Studies have found that estrogen can reduce insulin resistance, improve the ability to deal with glucose, so as to reduce blood glucose. So we used male mice in our research to avoid the effects of estrogen [19]. Age and weight matched db/m mice were used as control group. All experimental procedures were performed in accordance with the Guidelines for the Care and Use of Laboratory Animals (NIH Publication No. 85-23; revised 1996). The study was carried out in compliance with the ARRIVE guidelines as well as the relevant national laws on the protection of animals. All mice were maintained at room temperature with a relative humidity of ~ 60% environment with a 12 h light-dark cycle and fed normal food and water. After anesthesia and sacrifice with CO_2_, the SMG was removed immediately from the animal, frozen in liquid nitrogen for 1 min, and then stored at − 80 °C.

### RNA extraction and microarray analysis

Four mice were andomly chosen from each group. The total RNA was extracted from the SMG tissues using the TRIzol LS Reagent (Invitrogen, CA, USA), and was used for lncRNA microarray analysis (KangCheng, Shanghai, China). The concentration and purity of RNA were measured using a NanoDrop ND-1000 (Thermo, MA, USA), and quantity and integrity of RNA was tested using denaturing agarose gel electrophoresis. Microarray hybridization was performed using Quick Amp Labeling Kit, One-Color (Agilent, CA, USA) based on the manufacturer’s standard protocols. Agilent Feature Extraction software (Agilent, CA, USA) was used to analyze acquired array images. Quantile normalization and subsequent data processing were performed using the GeneSpring GX v12.1 software package (Agilent, CA, USA). All microarray hybridization and analyses were performed by KangChen Biotech (Shanghai, China). The accession number of microarray data was GSE141411.

### Real-time quantitative polymerase chain reaction (RT-qPCR)

We selected the 11 most significantly differentially expressed lncRNAs for RT-qPCR validation. Briefly, total RNA was extracted from two groups of SMG tissues using TRIzol reagent (Invitrogen, CA, USA). Total RNA (1 μg) was reverse-transcribed into cDNA using 5X All-In-One RT MasterMix (ABM, Richmond, BC, Canada) according to the manufacturer’s instructions. The RT-qPCR was performed on ViiA™ 7 Real-Time PCR System (Thermo, MA, USA) using a 2X SYBR Green qPCR Master Mix (Bimake, HOU, USA). The RT-qPCR primers were synthesized by Sangon Biotech (Shanghai, China). ΔΔCq method was used.

### Bioinformatics analysis

Volcano plot filtering was used to identify differentially expressed lncRNAs based on the threshold defined as fold-change > 2.0 (Student’s t test *P* < 0.05). The differentially expressed lncRNAs between db/db and db/m mice were displayed by hierarchical clustering. GO analysis (http://www.geneontology.org/) and KEGG analysis (http://www.genome.jp/kegg/) were used to explore the roles of the target genes of differentially expressed lncRNAs [20–22].

### Coding-noncoding co-expression and competing endogenous RNA networks

The normalized signal intensities of differentially expressed mRNA from whole-genome expression profiling (https://www.ncbi.nlm.nih.gov/geo/query/acc.cgi?acc=GSE141412) and differentially expressed lncRNA validated by RT-qPCR were used to construct CNC networks. In this study, lncRNAs, mRNAs, and miRNAs were collected from the same batch of samples. The lncRNA-mRNA pairs were identified based on a Pearson’s correlation coefficient of more than 0.95. The CNC network was constructed using Cytoscape software (The Cytoscape Consortium, CA, USA). Next, we constructed the lncRNA-miRNA-mRNA ceRNA networks based on microarray data. The lncRNA-miRNA interactions and miRNA-mRNA pairs were predicted using Arraystar’s home-made miRNA target prediction software and TargetScan & miRanda [23–26].

### Statistical analysis

Statistical analysis was performed by GraphPad Prism 5.0 (GraphPad Software, USA). Data of relative expression level of lncRNAs were expressed as the mean ± standard error. Student’s t-test was used to test whether the two sets of data were statistically significant, and *P* < 0.05 was considered to indicate statistical significance of gene expression difference between the two group.

## Results

### Expression profiling of lncRNAs in the SMG in db/db mice

**Fig. 1:**
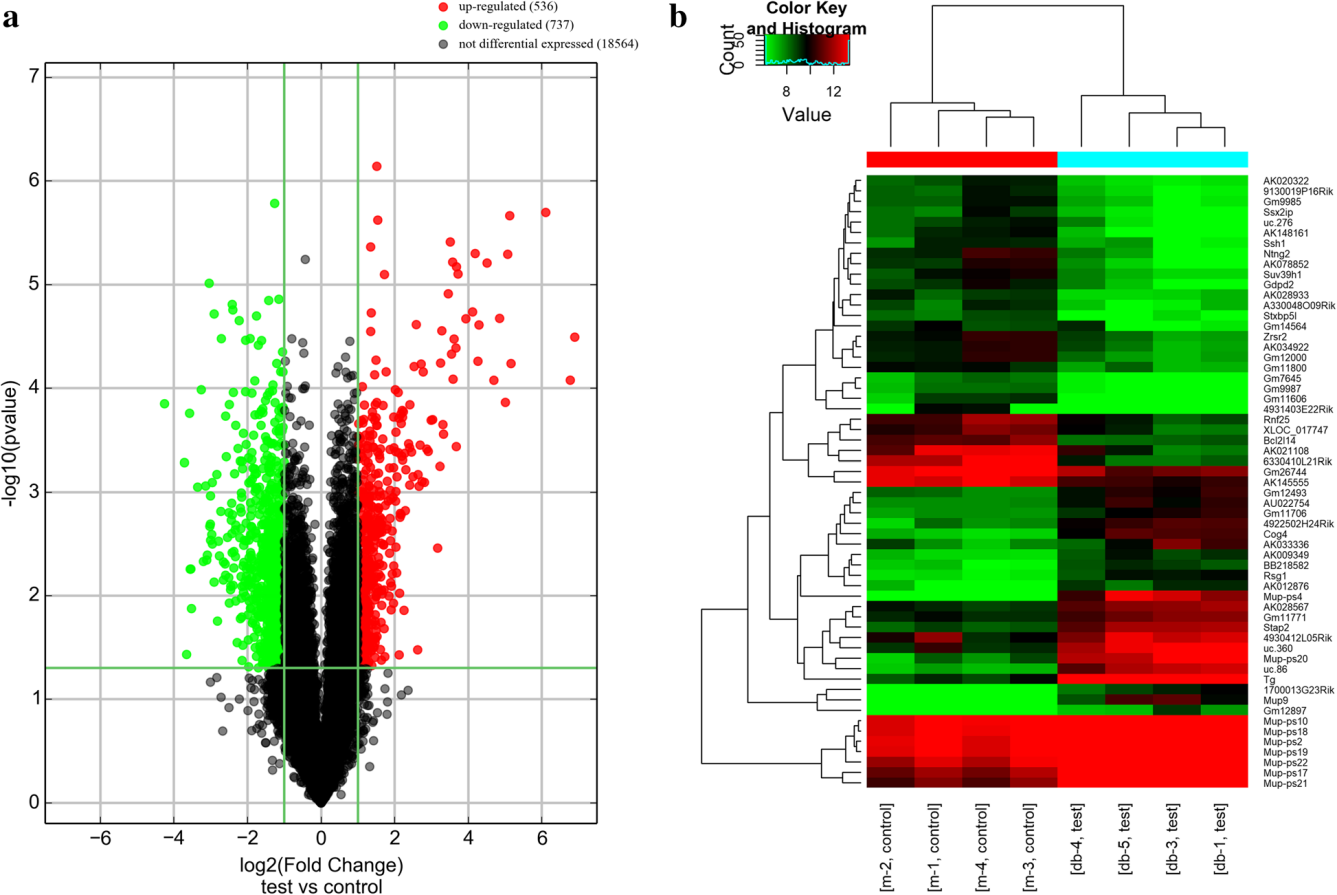
The expression profiling changes of significantly differentially expressed lncRNAs in db/db and db/m mice. **A** Volcano plots presenting differences in the expression of lncRNAs between db/db mice and db/m mice. **B** Heat maps showing the top 60-fold change distinct mRNAs expression profiles between the db/db mice and db/m mice

In our study, lncRNA expression profile of the SMG tissues was carried out using microarray analysis. In comparison with db/m mice, 1273 differentially expressed lncRNAs were identified in the SMG tissues from db/db mice, including 536 upregulated and 737 downregulated lncRNAs (fold-change *>* 2.0, *P <* 0.05). Table 1 provided the top ten upregulated and downregulated lncRNAs. A volcano plot of lncRNA expression profiles was displayed in Fig. 1A, the heat maps of the 60 most significantly differentially expressed lncRNAs (30 upregulated and 30 downregulated) was displayed in Fig. 1B.

**Table 1:** Top 10 upregulated and downregulated lncRNA in microarray

Probe Name	Regulation	Seqname	Gene Symbol	RNA length	Chrome	Fold Change	***P***-value
ASMM10P027186	up	ENSMUST00000137025	Mup9	787	chr4	118.8211935	3.21678E-05
ASMM10P055456	up	ENSMUST00000169784	Mup-ps4	474	chr4	109.195563	8.37692E-05
ASMM10P003383	up	ENSMUST00000163495	Tg	4952	chr15	68.820901	2.01088E-06
ASMM10P029469	up	ENSMUST00000120341	Gm12897	250	chr4	35.7783047	5.78685E-05
ASMM10P055944	up	TCONS_00006898	TCONS_00006898	75	chr12	34.9270569	2.16253E-06
ASMM10P058117	up	AI642987	humanlincRNA1158	226	chr2	33.6200933	5.09835E-06
ASMM10P027204	up	ENSMUST00000121132	Mup-ps20	389	chr4	32.1768747	0.000137217
ASMM10UP787	up	uc.86+	uc.86	340	chr2	28.8579697	2.12019E-05
ASMM10P002133	up	ENSMUST00000139794	1700013G23Rik	446	chr11	25.8974187	8.39099E-05
ASMM10P010990	up	AK078011	AK078011	742	chr14	22.7760404	6.19189E-06
ASMM10P026501	down	NR_040589	6330410L21Rik	3869	chr3	19.1022874	0.000141403
ASMM10P020728	down	NR_040496	Gad1os	905	chr2	13.125109	0.000521638
ASMM10P002832	down	ENSMUST00000151559	Uqcr10	510	chr11	12.6015112	0.036962683
ASMM10P048643	down	ENSMUST00000122825	Myo15	2285	chr11	11.9126698	0.000174835
ASMM10P010250	down	AK021108	AK021108	489	chr13	11.7795425	0.00555039
ASMM10P036325	down	ENSMUST00000120706	Gm11606	617	chr1	11.6265157	0.005532558
ASMM10P019099	down	NR_045306	4931403E22Rik	668	chr19	11.4948991	0.013329559
ASMM10P025520	down	AK076356	AK076356	2681	chr3	10.2321104	0.000895843
ASMM10P020729	down	ENSMUST00000150702	Gm16292	608	chr2	9.5576314	0.000103482
ASMM10P019262	down	TCONS_00016118	XLOC_012418	330	chr19	9.1857411	0.004675328

### Validation of lncRNA microarray results

**Fig. 2:**
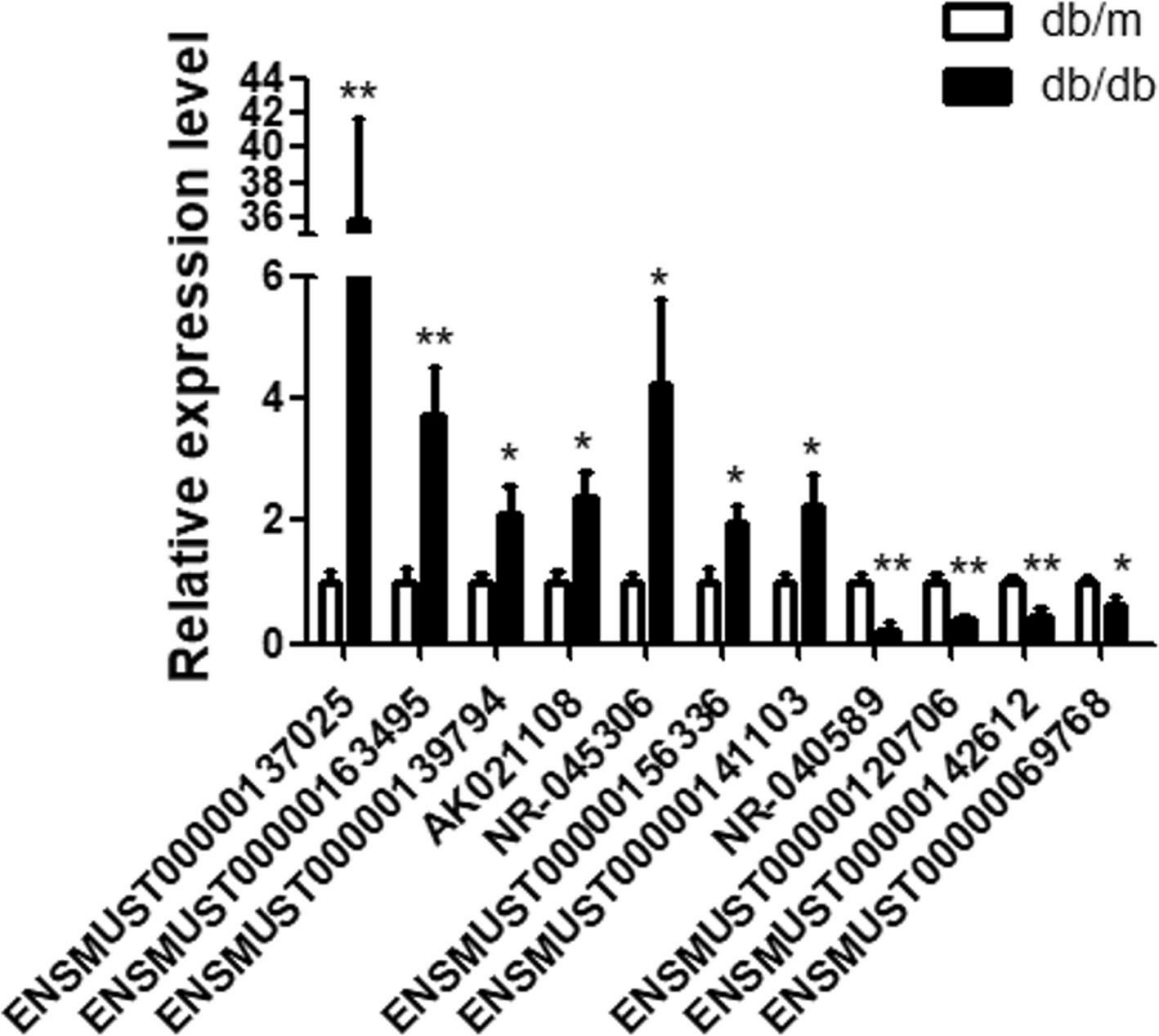
Validation of lncRNA expression. The relative expression levels of selected lncRNAs as detected by RT-qPCR. β-Actin was used as a housekeeping gene for normalizing changes in specific gene expression. *P < 0.05 and **P < 0.01 vs db/m mice, *n* = 6 ~ 9 per group. RT-PCR, real-time quantitative polymerase chain reaction

Because there is no previous research that has explored the relationship between lncRNA and saliva secretion in diabetes, there is no literature that can be referred to. Considering that the most differentially expressed lncRNAs should play an important role in the process, we chose the top 10 (up and down regulated) differentially expressed lncRNAs in our study [27]. In these 20 lncRNAs, 9 lncRNAs could not be verified by qRT-PCR because primers could not be designed. Finally, 11 lncRNAs (NR-040589, ENSMUST00000142612, ENSMUST00000139794, ENSMUST00000163495, ENSMUST00000137025, AK021108, NR-045306, ENSMUST00000156336, ENSMUST0000014113, ENSMUST00000120706 and ENSMUST0000069768) were selected from microarray results to carry out RT-qPCR. Of these, five lncRNAs, including 2 downregulated (NR_040589 and ENSMUST00000142612) and 3 upregulated (ENSMUST00000139794, ENSMUST00000163495, and ENSMUST00000137025), were consistent with microarray analysis results (Fig. 2). The advantages of microarray analysis were fast and efficient, but it also has false positives and false negatives. It is a common phenomenon that qRT-PCR and microarray results are inconsistent. Usually, we selected lncRNA with consistent result for in-depth research [28]. The corresponding primers used were listed in Table 2.

**Table 2:** The Primers of differentially expressed lncRNAs

LncRNA	Forward prime	Reverse primer
ENSMUST00000137025	TGCACAACTATGTGAGGAGCATGG	GGAGGCTCAGGCCATTCTTCATTC
ENSMUST00000163495	GCAATGAGGAGAGTGGAGCAACC	GTCTGCCAAGAGTCCGAAGTAACC
ENSMUST00000139794	GACTGGCAGGAAGAACGGATGAC	GGCACACCTGGTTCCATCTTAGAC
NR_040589	TCCCGCTTCTCTCGCTTCTCTC	CTGTTGCTCGCCTTCCTGCTG
AK021108	GGTGGCTGTGAAGACGCTGAAG	TTGACAATGTGCTCGTGCTGGAG
ENSMUST00000120706	AGGTGGCTGGTTTCTCTGGA	AGCAGCTACTCTTGCCTGGA
NR_045306	GCCTGCCTGCCTCCTCTCTC	TTCTGAGCCTGGGTGTCTCTGG
ENSMUST00000142612	TTGAGCATGTGCCAGACAGAAGTG	TCTCCATCGTGTTGCCTTCAAGTG
ENSMUST00000069768	ACGGACACCTCTCCTGCCTTG	CTTCCTGAAGCTCGCGGAGAATC
ENSMUST00000156336	ATGAGCGACTGAGCAGGACTACC	GGCTGGCGTCCTCACCTCTAG
ENSMUST00000141103	AACTGAGCAGAGACAGCGTGTG	TCATGGTCTTGTCGTGAGATGACG

### Coding-noncoding co-expression network analysis

**Fig. 3:**
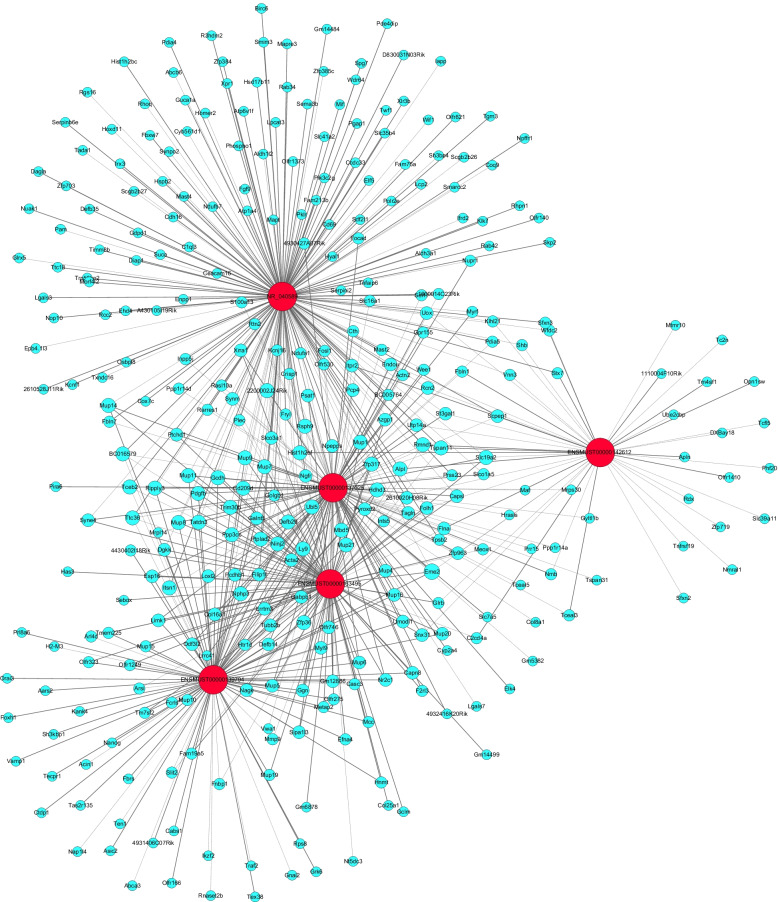
CNC network. Red nodes are lncRNAs, and blue nodes are mRNAs. Positive correlation is a solid line, and negative correlation is a dashed line

**Fig. 4:**
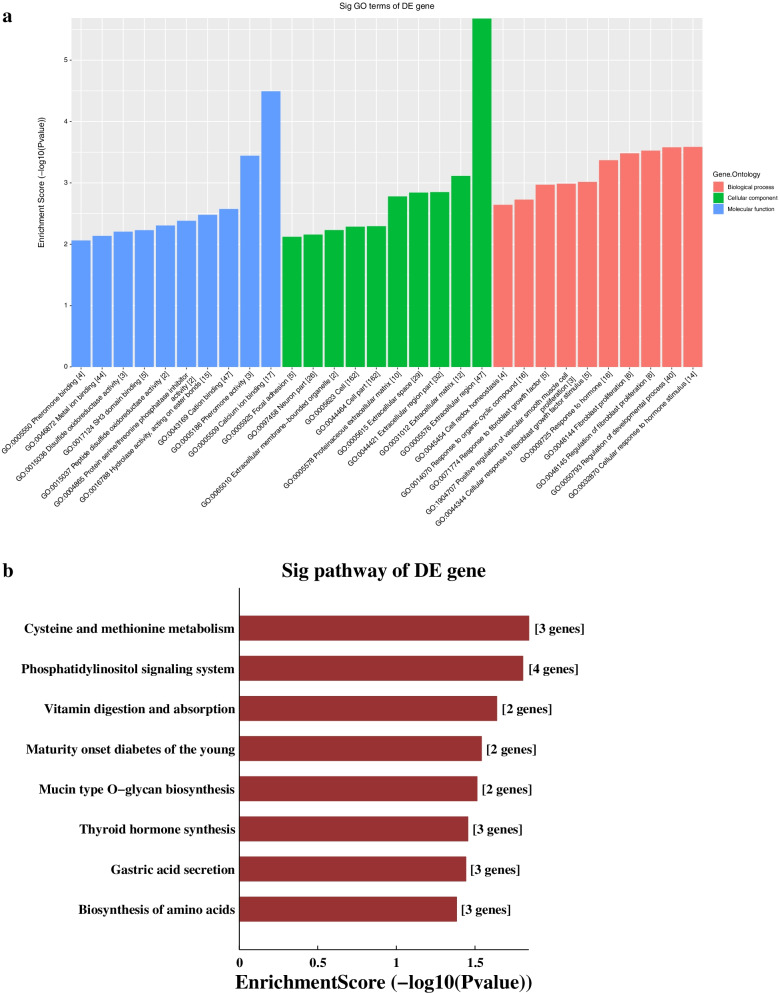
Bioinformatic analysis of CNC network. **A** GO and **B** KEGG pathway analyses based on CNC analysis results

Five lncRNAs validated by RT-qPCR and the differentially expressed mRNA from the database (GSE141412) was used to construct the CNC network shown in Fig. 3. There were 615 lncRNA-mRNA connection pairs in the network, including 369 positive connection pairs and 246 negative pairs. The top ten positive and negative interaction pairs according to the Pearson’s correlation coefficient were showed in Table 3. These closely interaction pairs may be involved in the regulation of saliva secretion of SMG in db/db mice. GO analysis based on the target genes of the CNC network showed that the mostly enriched biological process was “Cellular response to hormone stimulus”, the mostly enriched cellular component was “Extracellular region”, while mostly enriched molecular function was “Calcium ion binding”, as shown in Fig. 4A. KEGG pathway analysis revealed that the mainly enriched pathways were “Cysteine and methionine metabolism”, “Phosphatidylinositol signaling system”, and “Vitamin digestion and absorption”, as shown in Fig. 4B. Cystathionine-gamma-lyase (CTH), glutamate-cysteine ligase modifier subunit (Gclm) and phosphoserine aminotransferase 1 (PSAT1) were enriched in “Cysteine and methionine metabolism”. These enriched GO terms and pathways may participate in the DIO-induced hyposalivation.

**Table 3:** Top ten positive and negative correction pairs of lncRNA-mRNA

mRNA	LncRNA	Pearson’s correlation coefficient	Correlation type	***P***-value	False discovery rate
Mup11	ENSMUST00000163495	0.999661	+	9.75E-11	7.06E-06
Mup1	ENSMUST00000163495	0.998694	+	5.57E-09	0.000202
Mup14	ENSMUST00000163495	0.998433	+	9.60E-09	0.000232
Mup20	ENSMUST00000163495	0.998044	+	1.87E-08	0.000338
Mup8	ENSMUST00000163495	0.997129	+	5.90E-08	0.00078
Mup7	ENSMUST00000163495	0.997041	+	6.46E-08	0.00078
Defb29	ENSMUST00000139794	0.9964	+	1.16E-07	0.001203
Kcnj16	NR_040589	0.995913	+	1.70E-07	0.00154
Mup5	ENSMUST00000163495	0.995466	+	2.32E-07	0.001869
1600014C23Rik	NR_040589	0.995075	+	2.98E-07	0.002063
Acin1	ENSMUST00000139794	−0.95011	–	0.000299	0.035206
Zfp36	NR_040589	−0.95025	–	0.000297	0.035084
Mup7	NR_040589	−0.9503	–	0.000296	0.035024
Utp14a	ENSMUST00000142612	−0.95034	–	0.000295	0.035024
Scgb2b27	NR_040589	− 0.95037	–	0.000294	0.035024
Synm	ENSMUST00000163495	−0.95042	–	0.000293	0.035024
H2-M3	ENSMUST00000139794	−0.9505	–	0.000292	0.034963
Ppp1r14a	ENSMUST00000137025	−0.95051	–	0.000292	0.034963
Ube2cbp	ENSMUST00000142612	−0.95073	–	0.000288	0.034859
Ppp1r14d	ENSMUST00000137025	−0.95078	–	0.000287	0.034859

### Competing endogenous RNA network analysis

**Fig. 5:**
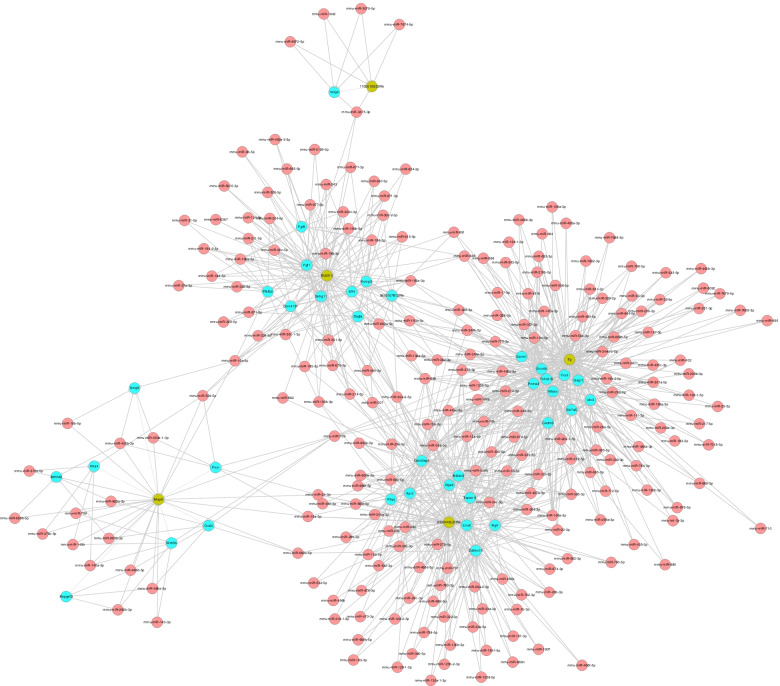
CeRNA network. Red circles represent miRNAs, blue circles represent mRNAs, and green circles represent lncRNAs

**Fig. 6:**
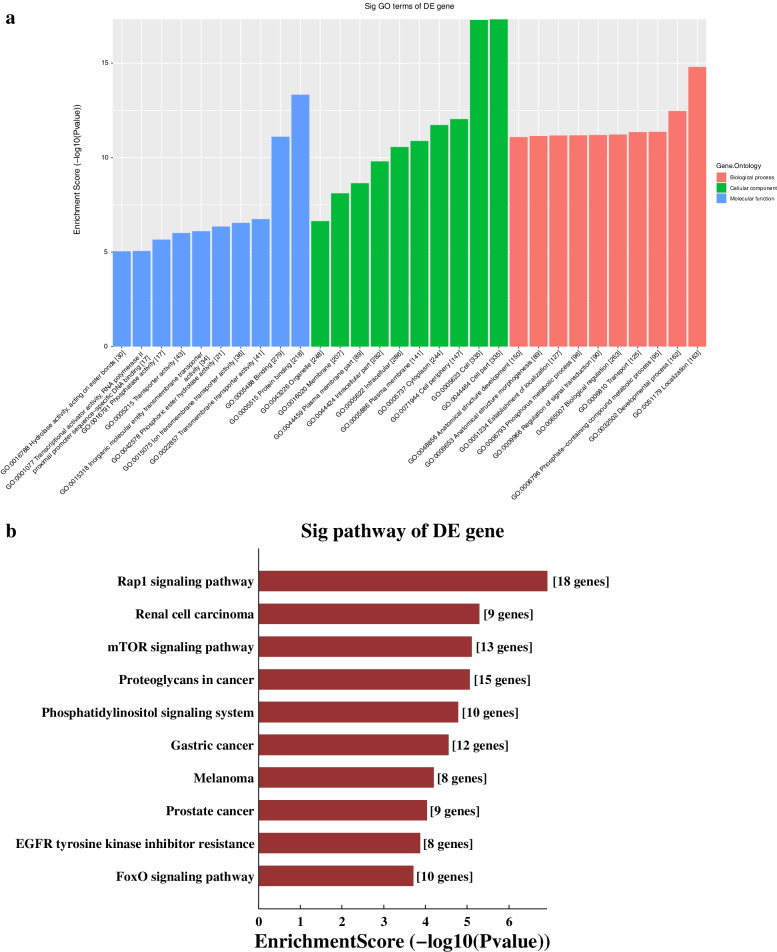
GO and KEGG pathway analyses of the ceRNA network. **A** GO and **C** KEGG pathway analyses based on the competing endogenous RNA analysis results

We constructed ceRNA network based on five lncRNAs validated using RT-qPCR and all differentially expressed mRNAs in the microarray (Fig. 5)*.* We totally found 37 mRNAs in the ceRNA network (Supplemental Table S1). GO analysis based on the target genes of ceRNA analysis results showed that the mostly enriched biological process was “Localization”, the mostly enriched cellular component was “Cell part”, and the mostly enriched molecular function was “Protein binding” (Fig. 6A). KEGG pathway analysis revealed that the main pathways included “Rap1 signaling pathway”, “Renal cell carcinoma” and the “mTOR signaling pathway” (Fig. 6B). Signal-induced proliferation-associated 1 like 3 (SIPA1L3), enabled homolog (ENAH), thrombospondin 1 (Thbs1) et al. were enriched in “Rap1 signaling pathway”. These results showed that biological processes and regulatory pathways might play vital roles in the secretion of SMG in obesity.

## Discussion

In humans, approximately 60% of resting saliva and 40% of stimulated saliva is secreted from the SMG [29]. However, studies focusing on the SMG in the context of diabetes are very limited. We collected SMG of mice for high-throughput sequencing to study the underlying mechanism by which diabetes reduced saliva secretion. Compared with db/m mice, 536 upregulated and 737 downregulated lncRNAs were identified, these changed lncRNAs may play an important role in the diabetes-induced hyposalivation.

We performed RT-qPCR verification on 11 differentially expressed lncRNAs with the most significant dysregulations, and five differentially expressed lncRNAs exhibited consistent with the results of high-throughput sequencing. We previously determined the whole genome expression profile of SMG tissues in db/db mouse and found that 1146 mRNAs exhibited significant dysregulation. Of these, 606 mRNAs were upregulated and 540 mRNAs were downregulated, and this data should be found in GSE141412. We used these five lncRNAs to perform CNC network and ceRNA network analyses in combination with 1146 differentially expressed mRNAs.

GO analysis of mRNA obtained from the CNC network showed that “Calcium ion binding” is highly enriched in the molecular function. The mobilization of Ca^2+^ played an important role in salivary secretion, the activation of muscarinic cholinergic receptors rapidly triggered the release of intracellular Ca^2+^ from the endoplasmic reticulum and subsequently the influx of Ca^2+^ from the extracellular medium, resulting in a sustained increase in intracellular Ca^2+^ [30, 31]. The increased intracellular Ca^2+^ induced the transport of aquaporin 5, leading to the formation of water pores and thus promoting the rapid increase in transcellular water permeability [32]. In addition, our previous study found that adiponectin can also induce salivary secretion of the rat db/db mouse by activating adenosine monophosphate-activated protein kinase, and the Ca^2+^ signaling pathway played an important role in this process [33]. In human and rabbit SMGs, the activation of muscarinic cholinergic receptors and transient receptor potential vanilloid subtype 1 increased salivary secretion via increased intracellular Ca^2+^ [34, 35]. In human transplanted epiphora SMG, the elevated intracellular Ca^2+^ mobilization induced by muscarinic acetylcholine receptors activation contributed to hypersecretion [34]. In spontaneously hypertensive rats, the damaged Ca^2+^ response to carbachol was confirmed in the acinar cells of spontaneously hypertensive rats, which may also be related to the reduced salivary secretion caused by hypertension [36]. These studies indicate that the increased intracellular Ca^2+^ derived from extracellular and intracellular Ca^2+^ plays an important role in the salivary secretion of SMG. Our results from microarray analysis show that significantly altered “calcium ion binding”, suggesting that the salivary secretion of the SMG may be also affected by the Ca^2+^ signaling pathway during diabetes. However, the regulatory mechanism of related lncRNA-mRNA requires further research.

KEGG analysis from the CNC network showed that “Cysteine and methionine metabolism” is the highest enriched pathway. An important feature of diabetes is abnormal nutrient homeostasis. One of metabolic abnormalities is to change the metabolism of sulfur-containing amino acid cysteine. Cysteine produced hydrogen sulfide through enzymatic decomposition, which is a gas transmitter regulating glucose and lipid homeostasis [37]. Studies found that type 2 diabetes was associated with lower plasma cysteine levels [38]. Both cysteine and hydrogen sulfide can inhibit β Cells release insulin after glucose stimulation. The level of low hydrogen sulfide found in plasma of patients with type 2 diabetes may be a compensatory response. In addition to regulating insulin secretion, hydrogen sulfide may regulate other aspects of cell physiology include viability and respiratory function of mitochondria. In the stretptozotocin-induced diabetes model, elevated hydrogen sulfide decrease the viability of cultured β Cells [39]. Our previous research found that high glucose (HG) induced mitochondrial dysfunction and PINK1/Parkin-mediated mitophagy in cultivated rat SMG cell line SMG-C6 cells [40]. We also demonstrated that autophagy plays a crucial role in HG-modulated aquaporin 5 degradation, which contributes to the dysfunction of diabetic SMG [7]. However, the mechanism of diabetes inducing mitophagy and mitochondrial dysfunction in salivary gland is still not well understood. Our results from microarray analysis suggested that the salivary secretion of the SMG may be also affected by “Cysteine and methionine metabolism” during diabetes. From these results, we speculated that these 5 validated lncRNAs might the major regulatory molecules.

LncRNA regulates mRNA via various mechanisms, one of which is ceRNA-mediated changes in the expression of downstream molecules regulated by miRNAs. We performed ceRNA network analysis on five significantly altered lncRNAs and 1146 dysregulated mRNAs to identify the related pathways regulated by the miRNA pathway. We also analyzed the obtained mRNAs via GO and KEGG analyses. KEGG analysis suggested that the “mTOR signaling pathway” was significantly enriched, suggesting that these five lncRNAs might affect the downstream “mTOR signaling pathway” through ceRNA. The PI3K/Akt /mTOR pathway is an intracellular signaling pathway that played a key role in regulating cell cycle-mediated processes, including cellular quiescence and cell proliferation [41], and various disease such as epithelial ovarian cancer [42]. Many studies have demonstrated that the “mTOR signaling pathway” plays an important role in the pathophysiological process of diabetes. In MC3T3-E1 cells, high glucose increased the production of reactive oxygen species and induced autophagy by inhibiting the Akt/mTOR pathway [43]. The mTOR/PI3K/Akt pathway was involved in the regulation of autophagy in diabetic kidney [44]. PI3K/Akt/mTOR pathway was significantly downregulated in the brain of streptozotocin-induced type 2 diabetic rats, this might explain the neurodegeneration commonly observed in diabetes [45]. In addition, the mTOR pathway is also related to the process of salivary secretion. Bleomycin induced epithelial to mesenchymal transformation of human SMG cells via the Akt/mTOR pathway [46]. In our previous studies, we found that autophagy induced aquaporin 5 degradation through the PI3K/Akt /mTOR pathway signaling pathway, resulting in decreased salivary secretion of SMG in db/db mice [7]. These studies suggest that the “mTOR signaling pathway” may play an important role in SMG injury caused by diabetes. The five lncRNAs we verified may involve in the regulation of the “mTOR signaling pathway” through the ceRNA mechanism via miRNA sponging, but further research is needed.

KEGG analysis from the ceRNA network also showed that “Rap1 signaling pathway” is the highest enriched pathway. Ras-associated protein1 (Rap1) is a member of the Ras-like small GTP binding protein family. In endothelium Rap1 is a key positive regulator of angiogenesis and an important regulator of endothelial barrier [47]. Constitutive activation of Rap1 has not only led to T cell anergy, but also inhibited autophagy and supported cancer progression through various oncogenic events [48]. In addition, Rap1 signaling has been shown to play a role in Ca^2+^ signaling pathways to control the important cellular functions. Rap1 modulates cardiac Ca^2+^ homeostasis through the regulation of Ca^2+^-induced Ca^2+^ release in adult ventricular cardiomyocytes [49]. Rap1 may act directly to open Ca^2+^-sensitive K^+^ channels, inducing smooth muscle hyperpolarization and leading to vasorelaxation. Rap1 plays an important role in maintaining normal vascular contractile state and contributes to blood pressure regulation by altering Ca^2+^ sensitivity of vascular smooth muscle cells [50]. As intracellular Ca^2+^ plays an important role in the salivary secretion of SMG, these 5 validated DE lncRNAs might be a novel treatment strategy for future treatment of diabetes-induced xerostomia in the clinic.

It is worth noting that both KEGG analyses from the ceRNA network and CNC network found “Phosphatidylinositol signaling system” was significantly enriched. Phosphatidylinositol is involved in many signal transduction pathways, such as PI3K/Akt pathway. PI3K/Akt signaling plays a central role in cellular physiology by mediating critical cellular processes, like glucose homeostasis. In db/db mice and high fat diet-induced diabetic mice, activates PI3K/Akt in an insulin-independent manner could attenuates hepatic gluconeogenesis and lipogenesis [51]. Zhang et al. found that artesunate and metformin combination improve salivary gland hypofunction in murine Sjögren’s syndrome though regulating the PI3K/Akt pathway [52]. We speculated that these 5 validated DE lncRNAs might be key molecules regulating PI3K/Akt pathway. CeRNA may be one of the regulatory mechanisms, and there might be other regulatory mechanisms. These 5 validated DE lncRNAs we found regulating PI3K/Akt pathway might be a novel treatment strategy for future treatment of diabetes-induced xerostomia in the clinic.

The present study has some potential limitations. First, the results of differential expression analysis were from small samples. Second, we have used db/db mice for our experiment. Db/db mouse was a model of spontaneous type 2 diabetes with leptin receptor-deficient. The expression levels of the identified differentially expressed lncRNAs may vary in case of human. In addition, the potentially biological functions of differentially expressed lncRNAs have not been validated by technical and biological experiments.

Our results revealed that lncRNAs may play an important role in SMG dysfunction caused by diabetes. CNC network and ceRNA network analyses showed that “calcium ion binding”, “mTOR signaling pathway” and other pathway potentially served as the downstream pathways regulated by the five lncRNAs in our study. Our findings expand our understanding of the roles of lncRNAs in salivary secretion in diabetic mice, and help us to understand the roles of lncRNAs in the pathogenesis of diabetes-induced hyposalivation by performing regulatory networks analyses. This study can provide valuable information for further research.

## Supplementary Information

**Additional file 1: Supplemental Table S1.** Analysis of ceRNA network.

## Data Availability

The datasets generated and/or analysed during the current study are available in the (Gene Expression Omnibus) repository, https://www.ncbi.nlm.nih.gov/geo/query/acc.cgi?acc=GSE141411.

## Abbreviations

LncRNA: Long non-coding RNA
miRNA: MicroRNA
SMG: Submandibular gland
RT-qPCR: Real-time quantitative polymerase chain reaction
mTOR: Mammalian target of rapamycin
GO: Gene Ontology
KEGG: Kyoto Encyclopedia of Genes and Genomes
CNC: Coding-noncoding co-expression
ceRNA: Competing endogenous RNA
PI3K: Phosphatidylinositol 3-kinase
Akt: Protein-serine-threonine kinase
