# Supplementary material for: Profiling the lncRNA-miRNA-mRNA interaction network in the submandibular gland of diabetic mice
Source: BMC Endocr Disord. 2022 Apr 21;22:109. doi: 10.1186/s12902-022-01019-1 (PMC9028094; doi:10.1186/s12902-022-01019-1)
Supplement: Supplementary file 1 — Additional file 1: Supplemental Table S1. Analysis of ceRNA network. [file 12902_2022_1019_MOESM1_ESM.docx]

Supplemental table S1. CeRNA network

| lncRNAs | | Common miRNAs | mRNAs | P values |
| --- | --- | --- | --- | --- |
| SeqName | GeneSymbol |  | CeSymbols |  |
| ENSMUST00000137025 | Mup9 | mmu-miR-15a-3p,mmu-miR-24-3p,mmu-miR-450a-1-3p,mmu-miR-450b-3p,mmu-miR-504-5p,mmu-miR-709 | Flcn | 0.000361141 |
|  |  | mmu-miR-143-3p,mmu-miR-145a-5p,mmu-miR-145b,mmu-miR-292b-3p,mmu-miR-709,mmu-miR-759 | Wnt5b | 0.002502078 |
|  |  | mmu-miR-486a-5p,mmu-miR-486b-5p,mmu-miR-669o-5p,mmu-miR-709,mmu-miR-883a-3p,mmu-miR-883b-3p | Orai3 | 0.003922103 |
|  |  | mmu-miR-292b-3p,mmu-miR-486a-5p,mmu-miR-486b-5p | Rapgef3 | 0.002651395 |
|  |  | mmu-miR-145a-5p,mmu-miR-145b,mmu-miR-376b-5p,mmu-miR-376c-5p,mmu-miR-504-5p | Htra4 | 6.82E-05 |
|  |  | mmu-miR-450a-1-3p,mmu-miR-450b-3p,mmu-miR-669b-5p,mmu-miR-883a-3p,mmu-miR-883b-3p | Mthfd2 | 0.004151659 |
|  |  | mmu-miR-10a-5p,mmu-miR-10b-5p,mmu-miR-450a-1-3p,mmu-miR-450b-3p | Smg9 | 0.006488549 |
| ENSMUST00000139794 | 1700013G23Rik | mmu-miR-1942,mmu-miR-3075-5p,mmu-miR-3475-3p,mmu-miR-6972-5p,mmu-miR-7674-5p | Vsig4 | 0.004622642 |
| ENSMUST00000142612 | Bcl2l14 | mmu-miR-185-3p,mmu-miR-188-3p,mmu-miR-30c-1-3p,mmu-miR-30c-2-3p,mmu-miR-329-3p,mmu-miR-351-5p,mmu-miR-362-3p,mmu-miR-485-3p,mmu-miR-615-5p,mmu-miR-670-5p,mmu-miR-691,mmu-miR-92a-2-5p | 9830107B12Rik | 2.35E-12 |
|  |  | mmu-miR-10a-5p,mmu-miR-185-3p,mmu-miR-193a-3p,mmu-miR-193b-3p,mmu-miR-207,mmu-miR-339-5p,mmu-miR-343,mmu-miR-344e-5p,mmu-miR-344h-5p,mmu-miR-546,mmu-miR-615-5p,mmu-miR-654-3p,mmu-miR-667-5p,mmu-miR-677-3p,mmu-miR-877-3p,mmu-miR-883a-5p | Kcnip3 | 8.23E-09 |
|  |  | mmu-miR-134-5p,mmu-miR-182-5p,mmu-miR-185-3p,mmu-miR-190b-3p,mmu-miR-193a-3p,mmu-miR-193b-3p,mmu-miR-194-2-3p,mmu-miR-199a-5p,mmu-miR-199b-5p,mmu-miR-204-5p,mmu-miR-211-5p,mmu-miR-214-5p,mmu-miR-302c-3p,mmu-miR-30c-1-3p,mmu-miR-30c-2-3p,mmu-miR-339-5p,mmu-miR-343,mmu-miR-3475-3p,mmu-miR-351-5p,mmu-miR-485-3p,mmu-miR-5129-5p,mmu-miR-5615-3p,mmu-miR-6367,mmu-miR-665-5p,mmu-miR-670-5p,mmu-miR-671-5p,mmu-miR-871-3p,mmu-miR-877-3p,mmu-miR-92a-2-5p | Fgf1 | 3.11E-06 |
|  |  | mmu-miR-193a-3p,mmu-miR-204-5p,mmu-miR-207,mmu-miR-211-5p,mmu-miR-214-5p,mmu-miR-302c-3p,mmu-miR-339-5p,mmu-miR-351-5p | Gm14137 | 1.62E-05 |
|  |  | mmu-miR-185-5p,mmu-miR-193a-3p,mmu-miR-193b-3p,mmu-miR-199a-5p,mmu-miR-199b-5p,mmu-miR-302c-3p,mmu-miR-320-5p,mmu-miR-329-5p,mmu-miR-546,mmu-miR-667-5p,mmu-miR-686,mmu-miR-871-3p,mmu-miR-877-3p,mmu-miR-883a-5p,mmu-miR-92a-2-5p | Elf4 | 2.79E-05 |
|  |  | mmu-miR-10a-5p,mmu-miR-182-5p,mmu-miR-188-3p,mmu-miR-190b-3p,mmu-miR-194-2-3p,mmu-miR-199a-5p,mmu-miR-199b-5p,mmu-miR-204-5p,mmu-miR-211-5p,mmu-miR-27a-5p,mmu-miR-31-5p,mmu-miR-351-5p | Pfkfb2 | 3.45E-05 |
|  |  | mmu-miR-10a-5p,mmu-miR-134-5p,mmu-miR-185-3p,mmu-miR-199a-5p,mmu-miR-199b-5p,mmu-miR-204-5p,mmu-miR-211-5p,mmu-miR-214-5p,mmu-miR-300-5p,mmu-miR-302c-3p,mmu-miR-30c-5p,mmu-miR-343,mmu-miR-485-3p,mmu-miR-504-5p,mmu-miR-546,mmu-miR-615-5p,mmu-miR-671-5p,mmu-miR-877-3p,mmu-miR-883a-5p,mmu-miR-92a-2-5p | Snhg11 | 3.55E-05 |
|  |  | mmu-miR-182-5p,mmu-miR-185-3p,mmu-miR-199a-5p,mmu-miR-199b-5p,mmu-miR-302c-3p,mmu-miR-351-5p,mmu-miR-450a-2-3p,mmu-miR-677-3p,mmu-miR-686,mmu-miR-96-5p | Fgf9 | 2.34E-06 |
|  |  | mmu-miR-185-5p,mmu-miR-329-3p,mmu-miR-344e-5p,mmu-miR-344h-5p,mmu-miR-351-5p,mmu-miR-362-3p,mmu-miR-670-5p,mmu-miR-882,mmu-miR-92a-2-5p | Cdc42ep4 | 1.42E-07 |
|  |  | mmu-miR-185-3p,mmu-miR-30c-1-3p,mmu-miR-30c-2-3p,mmu-miR-30c-5p,mmu-miR-344e-5p,mmu-miR-344h-5p,mmu-miR-351-5p,mmu-miR-615-5p,mmu-miR-670-5p,mmu-miR-671-5p,mmu-miR-92a-2-5p | Dagla | 3.38E-05 |
| ENSMUST00000163495 | Tg | mmu-miR-106a-3p,mmu-miR-134-3p,mmu-miR-141-3p,mmu-miR-15a-5p,mmu-miR-15b-5p,mmu-miR-16-5p,mmu-miR-17-3p,mmu-miR-183-5p,mmu-miR-193a-3p,mmu-miR-193b-3p,mmu-miR-195a-5p,mmu-miR-195b,mmu-miR-200a-3p,mmu-miR-20b-3p,mmu-miR-22-3p,mmu-miR-25-5p,mmu-miR-299a-3p,mmu-miR-299b-3p,mmu-miR-301a-5p,mmu-miR-322-5p,mmu-miR-326-3p,mmu-miR-328-3p,mmu-miR-330-5p,mmu-miR-370-3p,mmu-miR-497a-5p,mmu-miR-504-3p,mmu-miR-546,mmu-miR-665-3p,mmu-miR-673-5p,mmu-miR-686,mmu-miR-688,mmu-miR-709,mmu-miR-764-3p,mmu-miR-92a-1-5p | Nfasc | 3.70E-14 |
|  |  | mmu-miR-133a-5p,mmu-miR-15a-5p,mmu-miR-15b-5p,mmu-miR-16-5p,mmu-miR-195a-5p,mmu-miR-195b,mmu-miR-214-3p,mmu-miR-298-5p,mmu-miR-301a-5p,mmu-miR-322-5p,mmu-miR-328-3p,mmu-miR-33-5p,mmu-miR-331-3p,mmu-miR-337-3p,mmu-miR-344e-5p,mmu-miR-344h-5p,mmu-miR-345-5p,mmu-miR-361-3p,mmu-miR-3971,mmu-miR-449c-5p,mmu-miR-465a-3p,mmu-miR-465b-3p,mmu-miR-465c-3p,mmu-miR-485-5p,mmu-miR-497a-5p,mmu-miR-665-3p,mmu-miR-688,mmu-miR-883a-5p,mmu-miR-93-3p | Cry2 | 1.73E-08 |
|  |  | mmu-miR-133b-5p,mmu-miR-134-3p,mmu-miR-135b-3p,mmu-miR-145a-3p,mmu-miR-15a-5p,mmu-miR-15b-5p,mmu-miR-16-5p,mmu-miR-187-5p,mmu-miR-195a-5p,mmu-miR-195b,mmu-miR-214-3p,mmu-miR-322-5p,mmu-miR-329-3p,mmu-miR-344e-5p,mmu-miR-344h-5p,mmu-miR-361-3p,mmu-miR-362-3p,mmu-miR-486a-3p,mmu-miR-486b-3p,mmu-miR-497a-5p,mmu-miR-504-3p,mmu-miR-543-5p,mmu-miR-653-3p,mmu-miR-664-5p,mmu-miR-665-3p,mmu-miR-669h-5p,mmu-miR-691,mmu-miR-705 | Scn4b | 4.23E-11 |
|  |  | mmu-miR-133a-5p,mmu-miR-134-3p,mmu-miR-141-3p,mmu-miR-15a-5p,mmu-miR-15b-5p,mmu-miR-16-5p,mmu-miR-193a-5p,mmu-miR-195a-5p,mmu-miR-195b,mmu-miR-200a-3p,mmu-miR-298-5p,mmu-miR-322-5p,mmu-miR-326-3p,mmu-miR-330-5p,mmu-miR-337-3p,mmu-miR-344d-2-5p,mmu-miR-344e-5p,mmu-miR-344h-5p,mmu-miR-34a-5p,mmu-miR-34b-5p,mmu-miR-34c-5p,mmu-miR-361-3p,mmu-miR-370-3p,mmu-miR-449a-5p,mmu-miR-449b,mmu-miR-449c-5p,mmu-miR-497a-5p,mmu-miR-505-5p,mmu-miR-673-5p,mmu-miR-686,mmu-miR-695,mmu-miR-705,mmu-miR-712-5p,mmu-miR-764-3p,mmu-miR-876-5p,mmu-miR-92a-1-5p,mmu-miR-93-3p | Tnfrsf19 | 2.73E-09 |
|  |  | mmu-miR-134-3p,mmu-miR-145a-3p,mmu-miR-201-3p,mmu-miR-219b-5p,mmu-miR-28a-5p,mmu-miR-298-5p,mmu-miR-326-5p,mmu-miR-337-3p,mmu-miR-344d-2-5p,mmu-miR-344e-5p,mmu-miR-344h-5p,mmu-miR-485-5p,mmu-miR-486a-3p,mmu-miR-486b-3p,mmu-miR-664-5p,mmu-miR-665-3p,mmu-miR-669h-5p,mmu-miR-705,mmu-miR-708-5p,mmu-miR-709,mmu-miR-770-3p | Sarm1 | 5.70E-07 |
|  |  | mmu-miR-145a-3p,mmu-miR-187-5p,mmu-miR-19b-1-5p,mmu-miR-19b-2-5p,mmu-miR-298-5p,mmu-miR-326-3p,mmu-miR-329-3p,mmu-miR-331-3p,mmu-miR-337-3p,mmu-miR-344d-2-5p,mmu-miR-346-5p,mmu-miR-34a-5p,mmu-miR-34b-5p,mmu-miR-34c-5p,mmu-miR-361-3p,mmu-miR-362-3p,mmu-miR-449a-5p,mmu-miR-449b,mmu-miR-449c-5p,mmu-miR-504-3p,mmu-miR-546,,mmu-miR-653-3p,mmu-miR-664-5p,mmu-miR-7218-5p,mmu-miR-7669-3p,mmu-miR-7679-5p,mmu-miR-7682-3p,mmu-miR-7688-5p,mmu-miR-8092,mmu-miR-93-3p | Gsg1l | 1.49E-09 |
|  |  | mmu-let-7g-3p,mmu-miR-15a-5p,mmu-miR-15b-5p,mmu-miR-16-5p,mmu-miR-195a-5p,mmu-miR-195b,mmu-miR-298-5p,mmu-miR-299a-3p,mmu-miR-322-5p,mmu-miR-326-5p,mmu-miR-331-3p,mmu-miR-338-3p,mmu-miR-346-5p,mmu-miR-34a-5p,mmu-miR-34b-5p,mmu-miR-34c-5p,mmu-miR-432,mmu-miR-449a-5p,mmu-miR-449b,mmu-miR-449c-5p,mmu-miR-451b,mmu-miR-497a-5p,mmu-miR-504-3p,mmu-miR-665-3p,mmu-miR-669h-5p,mmu-miR-680,mmu-miR-705 | Cadm3 | 1.44E-08 |
|  |  | mmu-miR-194-1-3p,mmu-miR-217-5p,mmu-miR-298-5p,mmu-miR-301a-5p,mmu-miR-337-3p,mmu-miR-34a-5p,mmu-miR-34b-5p,mmu-miR-34c-5p,mmu-miR-361-3p,mmu-miR-449a-5p,mmu-miR-449b,mmu-miR-449c-5p,mmu-miR-451b,mmu-miR-455-3p,mmu-miR-505-5p,mmu-miR-546,mmu-miR-653-3p,mmu-miR-664-5p,mmu-miR-666-3p,mmu-miR-669h-5p,mmu-miR-673-5p,mmu-miR-764-3p,mmu-miR-92a-1-5p | Slc7a5 | 9.26E-08 |
|  |  | mmu-miR-15a-5p,mmu-miR-15b-5p,mmu-miR-16-5p,mmu-miR-195a-5p,mmu-miR-195b,mmu-miR-212-5p,mmu-miR-214-3p,mmu-miR-219b-5p,mmu-miR-22-3p,mmu-miR-322-5p,mmu-miR-326-5p,mmu-miR-344e-5p,mmu-miR-344h-5p,mmu-miR-346-5p,mmu-miR-370-3p,mmu-miR-451b,mmu-miR-484,mmu-miR-497a-5p,mmu-miR-504-3p,mmu-miR-543-5p,mmu-miR-653-3p,mmu-miR-673-5p | Pnma3 | 8.21E-08 |
|  |  | mmu-miR-145a-3p,mmu-miR-15a-5p,mmu-miR-16-5p,mmu-miR-195a-5p,mmu-miR-195b,mmu-miR-28a-5p,mmu-miR-322-5p,mmu-miR-337-3p,mmu-miR-432,mmu-miR-497a-5p,mmu-miR-504-3p,mmu-miR-505-5p,mmu-miR-665-3p,mmu-miR-708-5p,mmu-miR-710,mmu-miR-876-5p,mmu-miR-93-3p | Ubr2 | 7.92E-08 |
| NR_040589 | 6330410L21Rik | mmu-miR-125b-2-3p,mmu-miR-15a-5p,mmu-miR-15b-5p,mmu-miR-16-5p,mmu-miR-195a-5p,mmu-miR-195b,,mmu-miR-214-3p,mmu-miR-214-5p,mmu-miR-23a-5p,mmu-miR-24-3p,mmu-miR-322-3p,mmu-miR-322-5p,mmu-miR-326-3p,mmu-miR-330-5p,mmu-miR-466k,,mmu-miR-497a-5p,mmu-miR-582-3p,mmu-miR-673-5p,mmu-miR-760-3p,mmu-miR-761,mmu-miR-92a-2-5p | Lhx6 | 1.70E-10 |
|  |  | mmu-miR-125b-2-3p,mmu-miR-15a-5p,mmu-miR-15b-5p,mmu-miR-16-5p,mmu-miR-195a-5p,mmu-miR-195b,mmu-miR-214-3p,mmu-miR-214-5p,mmu-miR-23a-5p,mmu-miR-24-3p,mmu-miR-322-3p,mmu-miR-322-5p,mmu-miR-326-3p,mmu-miR-330-5p,mmu-miR-466d-5p,mmu-miR-466i-5p,mmu-miR-466k,mmu-miR-466l-5p,mmu-miR-497a-5pmmu-miR-582-3p,mmu-miR-669n,mmu-miR-673-5p,mmu-miR-760-3p,mmu-miR-761,mmu-miR-92a-2-5p | Lhx6 | 1.70E-10 |
|  |  | mmu-miR-128-1-5p,mmu-miR-128-2-5p,mmu-miR-15a-3p,mmu-miR-15b-5p,mmu-miR-195b,mmu-miR-207,mmu-miR-214-3p,mmu-miR-22-3p,mmu-miR-24-3p,mmu-miR-27b-5p,mmu-miR-34a-5p,mmu-miR-34b-5p,mmu-miR-34c-5p,mmu-miR-449a-5p,mmu-miR-449b,mmu-miR-449c-5p,mmu-miR-466d-5p,mmu-miR-466k,mmu-miR-470-3p,mmu-miR-497a-5p,mmu-miR-532-3p,mmu-miR-546,mmu-miR-669a-5p,mmu-miR-669f-5p,mmu-miR-669p-5p,mmu-miR-760-3p,mmu-miR-761 | Itpr2 | 3.47E-09 |
|  |  | mmu-miR-103-3p,mmu-miR-107-3p,mmu-miR-1258-5p,mmu-miR-130b-5p,mmu-miR-135a-1-3p,mmu-miR-15a-5p,mmu-miR-15b-5p,mmu-miR-1907,mmu-miR-1941-5p,mmu-miR-195a-5p,mmu-miR-214-3p,mmu-miR-22-3p,mmu-miR-23a-5p,mmu-miR-26a-2-3p,mmu-miR-26b-3p,mmu-miR-27b-5p,mmu-miR-28b,mmu-miR-28c,mmu-miR-322-3p,mmu-miR-322-5p,mmu-miR-370-3p,mmu-miR-380-5p,mmu-miR-497a-5p,mmu-miR-505-5p,mmu-miR-669k-5p,mmu-miR-669o-5p,mmu-miR-761,mmu-miR-92a-2-5p | Zdhhc15 | 2.30E-07 |
|  |  | mmu-miR-15a-5p,mmu-miR-15b-5p,mmu-miR-16-5p,mmu-miR-195a-5p,mmu-miR-195b,mmu-miR-204-3p,mmu-miR-207,mmu-miR-28b,mmu-miR-28c,mmu-miR-322-5p,mmu-miR-326-3p,mmu-miR-330-5p,mmu-miR-331-3p,mmu-miR-383-3p,mmu-miR-497a-5p,mmu-miR-532-3p,mmu-miR-669a-5p,mmu-miR-669f-5p,mmu-miR-669l-5p,mmu-miR-669p-5p,mmu-miR-702-3p | Nt5dc3 | 3.90E-08 |
|  |  | mmu-miR-138-5p,mmu-miR-212-5p,mmu-miR-214-3p,mmu-miR-22-3p,mmu-miR-26a-2-3p,mmu-miR-26b-3p,mmu-miR-28b,mmu-miR-28c,mmu-miR-331-3p,mmu-miR-34a-3p,mmu-miR-34a-5p,mmu-miR-34b-5p,mmu-miR-34c-5p,mmu-miR-370-3p,mmu-miR-449a-5p,mmu-miR-449b,mmu-miR-449c-5p,mmu-miR-546,mmu-miR-760-3p,mmu-miR-761,mmu-miR-874-3p,mmu-miR-880-5p,mmu-miR-92a-2-5p | Tspan11 | 7.82E-07 |
|  |  | mmu-miR-15a-5p,mmu-miR-15b-5p,mmu-miR-16-5p,mmu-miR-195a-5p,mmu-miR-195b,mmu-miR-1b-3p,mmu-miR-204-3p,mmu-miR-207,mmu-miR-214-3p,mmu-miR-29a-3p,mmu-miR-29b-3p,mmu-miR-29c-3p,mmu-miR-322-5p,mmu-miR-326-3p,mmu-miR-330-5p,mmu-miR-331-3p,mmu-miR-344g-3p,mmu-miR-370-3p,mmu-miR-485-3p,mmu-miR-497a-5p,mmu-miR-505-5p,mmu-miR-546,mmu-miR-673-5p,mmu-miR-702-3p,mmu-miR-761,mmu-miR-878-3p,mmu-miR-92a-1-5p | Rgs8 | 1.06E-05 |
|  |  | mmu-miR-15a-3p,mmu-miR-15a-5p,mmu-miR-15b-5p,mmu-miR-16-5p,mmu-miR-195a-5p,mmu-miR-195b,mmu-miR-214-3p,mmu-miR-322-3p,mmu-miR-322-5p,mmu-miR-326-3p,mmu-miR-330-5p,mmu-miR-383-3p,mmu-miR-466d-5p,mmu-miR-466i-5p,mmu-miR-466k,mmu-miR-497a-5p,mmu-miR-505-5p,mmu-miR-673-5p,mmu-miR-700-5p,mmu-miR-758-5p,mmu-miR-760-3p,mmu-miR-761,mmu-miR-874-3p,mmu-miR-92a-1-5p,mmu-miR-92a-2-5p | Ngfr | 1.44E-08 |
|  |  | mmu-miR-190b-3p,mmu-miR-204-3p,mmu-miR-214-3p,mmu-miR-218-1-3p,mmu-miR-29a-3p,mmu-miR-29b-3p,mmu-miR-29c-3p,mmu-miR-344g-3p,mmu-miR-34a-5p,mmu-miR-34c-5p,mmu-miR-449a-5p,mmu-miR-449b,mmu-miR-449c-5p,mmu-miR-495-3p,mmu-miR-496b,mmu-miR-532-3p,mmu-miR-532-5p,mmu-miR-546,mmu-miR-582-3p,mmu-miR-760-3p,mmu-miR-761,mmu-miR-880-5p,mmu-miR-92a-2-5p | Rbpj | 4.48E-07 |
|  |  | mmu-miR-138-5p,mmu-miR-15a-5p,mmu-miR-15b-5p,mmu-miR-16-5p,mmu-miR-195a-5p,mmu-miR-195b,mmu-miR-214-3p,mmu-miR-28b,mmu-miR-28c,mmu-miR-326-3p,mmu-miR-330-5p,mmu-miR-344g-3p,mmu-miR-34a-5p,mmu-miR-34b-5p,mmu-miR-34c-5p,mmu-miR-370-3p,mmu-miR-383-3p,mmu-miR-449a-5p,mmu-miR-449b,mmu-miR-449c-5p,mmu-miR-466d-5p,mmu-miR-466k,mmu-miR-495-3p,mmu-miR-505-5p,mmu-miR-669a-5p,mmu-miR-669f-5p,mmu-miR-669l-5p,mmu-miR-669p-5p,mmu-miR-761,mmu-miR-92a-2-5p | Cdc42ep4 | 3.95E-09 |
